# Excitation Light Dose Engineering to Reduce Photo-bleaching and Photo-toxicity

**DOI:** 10.1038/srep30892

**Published:** 2016-08-03

**Authors:** Colton Boudreau, Tse-Luen (Erika) Wee, Yan-Rung (Silvia) Duh, Melissa P. Couto, Kimya H. Ardakani, Claire M. Brown

**Affiliations:** 1 Department of Physiology, McGill University, Montreal, QC, Canada; 2 Advanced BioImaging Facility (ABIF), McGill University, 3649 Prom. Sir William Osler, Bellini Building Rm137, Montreal, QC, Canada, H3G 0B1; 3 Department of Anatomy and Cell Biology, McGill University, Montreal, QC, Canada; 4 Department of Microbiology and Immunology, McGill University, Montreal, QC, Canada.

## Abstract

It is important to determine the most effective method of delivering light onto a specimen for minimal light induced damage. Assays are presented to measure photo-bleaching of fluorophores and photo-toxicity to living cells under different illumination conditions. Turning the light off during part of the experimental time reduced photo-bleaching in a manner proportional to the time of light exposure. The rate of photo-bleaching of EGFP was reduced by 9-fold with light pulsing on the micro-second scale. Similarly, in living cells, rapid line scanning resulted in reduced cell stress as measured by mitochondrial potential, rapid cell protrusion and reduced cell retraction. This was achieved on a commercial confocal laser scanning microscope, without any compromise in image quality, by using rapid laser scan settings and line averaging. Therefore this technique can be implemented broadly without any software or hardware upgrades. Researchers can use the rapid line scanning option to immediately improve image quality on fixed samples, reduce photo-bleaching for large high resolution 3D datasets and improve cell health in live cell experiments. The assays developed here can be applied to other microscopy platforms to measure and optimize light delivery for minimal sample damage and photo-toxicity.

Live cell imaging has become common practice across the physical, life and health sciences. In light of this, many high quality reviews, procedures and protocols for live cell imaging have been published^1^,^2^,^3^,^4^,^5^,^6^,^7^,^8^,^9^,^10^. Fluorescent protein fusions and cellular markers are required to follow fundamental biological processes, visualize whole cells and/or proteins of interest. The very nature of the photo-physical process in the excitation of a fluorophore and emission of fluorescent light often leads to the secondary effects of photo-bleaching and photo-toxicity. However, a recent editorial piece highlighted how photo-toxicity has essentially been ignored by most researchers^11^. In fact, Carlton *et al*. showed that yeast cells “appeared normal” during fluorescence microscopy experimentation, but when observed twenty-four hours later it was shown that the cells were no longer undergoing proper cell division^12^. Wäldchen *et al*. demonstrated how 405 nm light (often used for live cell super-resolution microscopy) is much more photo-toxic to cells than lower energy 640 nm light^13^. These studies clearly demonstrate that live cell fluorescence imaging, even with low excitation doses, is more invasive than anticipated. Many live cell articles have focused on reducing light exposure^9^, and a recent review by Magidson and Khodjakov offers many helpful and practical solutions that can be taken into consideration to reduce photo-damage^14^. Manders *et al*. devised a clever solution called “controlled-light exposure microscopy” to reduce photo-bleaching and photo-toxicity by applying variable amounts of excitation light across the sample, depending on how much fluorescence is in a given spatial location^15^.

Another method to combat photo-bleaching and photo-toxicity is to deplete the sample of oxygen so that triplet state molecules cannot interact with oxygen and generate toxic reactive oxygen species. Reagents that remove dissolved oxygen reduce photo-bleaching in fluorescence imaging applications of anaerobic organisms such as bacteria^16^. In fact, most microscope mounting media for fixed samples contain oxygen scavengers such as anti-fade agents to reduce photo-bleaching of fluorescent probes. However, this is not an ideal solution for imaging of living mammalian cells or tissues that require an oxygen rich environment.

As a substitute for oxygen depletion it has been suggested that pulsing excitation light results in a dramatic reduction in photo-toxicity^17^,^18^,^19^. For example, delivering more frequent pulses of lower energy multi-photon laser light rather than one pulse of higher energy can improve light output and decrease photo-bleaching and photo-damage^20^. Theory has also been developed to suggest that scanning a confocal laser at high speed (e.g. resonant laser scanning confocal) should increase fluorescence yield and reduce photo-bleaching^21^. During the fluorescence excitation process when fluorophores enter the excited singlet state there is a finite, albeit low, probability that they will enter the “forbidden” triplet state. The triplet state, often called the “dark-state”, is long lived as it decays on the microsecond time scale versus the nanosecond timescale of the singlet state decay. If molecules in the triplet state absorb additional photons they have enough energy to break covalent bonds. These chemical reactions involve oxygen and can result in the release of reactive oxygen species. Highly reactive oxygen radicals can then cause further photo-bleaching and photo-damage, and cause photo-toxicity in living samples. In studies with pulsed LED illumination, a comparison was made between continuous or pulsed illumination of the sample. However, some of these experiments were carried out in such a manner that it is difficult to distinguish between reduced photo-toxicity due to an overall reduction in light exposure and reduced photo-toxicity specifically due to the pulsing of the light source^17^,^19^.

Donnert *et al*. demonstrated that slow pulsing of high power lasers with a 2 μs delay between pulses resulted in a 5–25-fold increase in fluorescence yield for green fluorescent protein (GFP) and Atto532 dyes^18^. The triplet state lifetime of one of the early mutants of wild-type GFP (S65T) was measured to be on the order of 2–25 μs^22^,^23^ and the lifetime for enhanced GFP (EGFP) has been reported as 4 μs^24^. Therefore, this delay between pulses likely provides enought time for the triple state molecules to relax and return to the ground state. This would probably decrease the likelihood that a molecule could absorb additional photons, break down and cause photo-damage and bleaching. Reduced bleaching with microsecond pulses of light therefore likely results in increased fluorescence yields because the molecules can undergo repeated excitation and fluorescence emission cycles before photo-destruction occurs.

In this study, continuous and pulsed confocal laser light on the nanosecond and microsecond time scales was applied to fixed and live samples to investigate the effects of light pulsing on photo-bleaching of fluorescent proteins and photo-toxicity to live cells. The same total light exposure was used to isolate the effect of the light pulse from the total amount of light exposure. Most life sciences researchers do not have access to microscopes with pulsed light sources, however they do typically have access to a standard confocal laser scanning microscope (CLSM). The rapid line scanning is a standard feature on the CLSM and it was used as a method to apply microsecond “pulses” of laser light to the specimen. Applying light on the microsecond timescale had no impact on image quality, but reduced photo-bleaching of EGFP 9-fold and dramatically reduced photo-toxicity. Photo-bleaching of mCherry was reduced 2-fold. The effective “pulsing” of light by the spinning disk confocal was also shown to reduce photo-bleaching of EGFP 2-fold. Our results suggest that the photo-bleaching and photo-toxicity depend on excited triplet state molecular destruction and the generation of oxygen radicals as the photo-bleaching is negated with the application of an oxygen scavenger. This is a novel but straightforward technique that researchers can immediately implement in the CLSM software without any need for hardware upgrades. It should be used to reduce photo-damage to fixed samples and photo-toxicity to live samples.

## Results

### Laser Pulsing on the nanoseconds time scale decreases photo-bleaching

**Figure 1:**
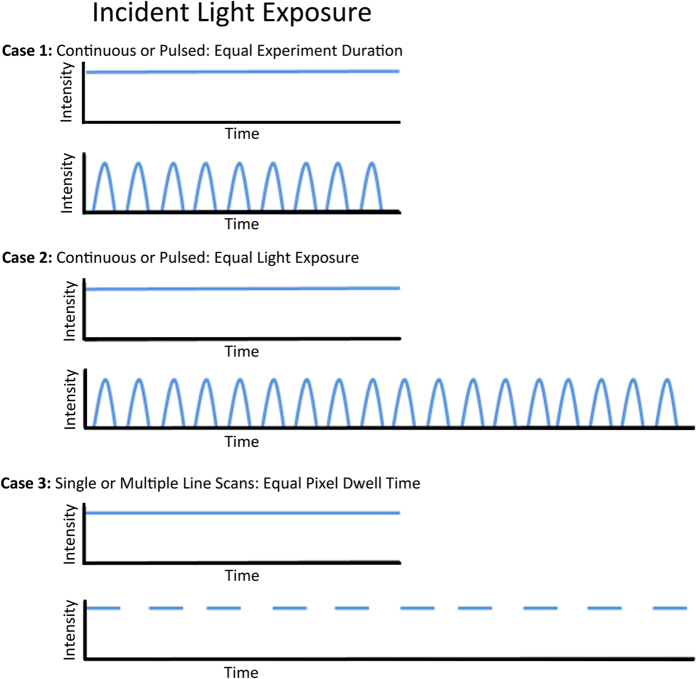
Nanosecond Pulsed Laser Experiments. **Case 1:** Equal experiment duration with continuous or pulsed laser excitation of equal peak power. **Case 2:** Equal total light exposure was created by exposing the sample longer if the light source was pulsed but keeping the peak light power constant. The experimental time was thus longer for pulsed light experiments but the total time the sample was exposed to light was constant. **Case 3:** Equal total light exposure was created by exposing the sample with single line scans and a long pixel dwell time or multiple line scans with a short pixel dwell time. The peak power and the total light exposure at each pixel location were kept constant.

**Figure 2:**
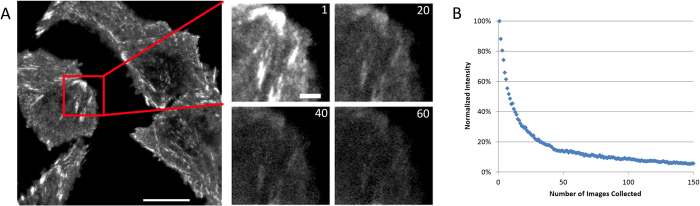
Photo-bleaching Experiments. (**A**) Fixed CHO-K1 cells expressing paxillin-EGFP were photo-bleached with 100–200 scans of the CLSM in 100 × 100 pixel regions of interest (ROIs). The zoomed in ROI shows how the fluorescent intensity bleaches over time. Frame numbers are shown in the upper right corner. (**B**) Intensity decay in an ROI shown as a function of the number of images collected. Scale bar is 10 μm for the whole cell image and 2 μm for the ROI images.

**Figure 3:**
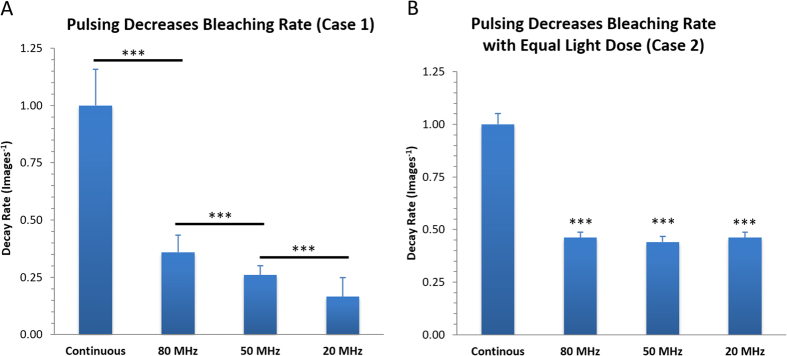
Rapid pulsing on the ns timescale. CHO-K1 cells expressing paxillin-EGFP were fixed and (**A**) imaged continuously with constant illumination at 40% laser power or with laser pulsing at 80, 50 or 20 MHz. The pixel dwell time and thus the total experiment time was held constant. Normalized decay rates are shown in units of images^−1^. (**B**) Normalized photobleaching decay rates when the pixel dwell time was varied to keep the total light exposure at each location within the sample constant. A two-tail t-test of two samples with unequal variances was used to calculate P values. There is no significant difference between 80, 50 and 20 MHz in B. Three stars (***) corresponds to a P values of <0.001. In panel (**B**) significance is relative to continuous laser illumination in all cases. Error bars are Standard Error of the Mean (SEM).

As stated in the introduction, many of the papers published on pulsed excitation, photo-bleaching and photo-toxicity introduced pulsing of the light source but kept the total experiment time the same. Thus, the total light dosage at the sample was decreased when moving from continuous light exposure to pulsed light. It is not surprising to see reduced photo-bleaching and photo-toxicity with reduced light levels. This scenario is shown as Case 1 in Fig. 1. Similar experiments using fixed CHO-K1 cells expressing paxillin-EGFP and mounted in PBS were conducted. The laser power of a 473 nm laser on the CLSM was held constant at 40% and the bleaching decay rate was measured during continuous imaging of a 100 × 100 pixel (8.7 μm × 8.7 μm) region of interest (ROI) of the sample (Fig. 2A). The fluorescence intensity decay curves (similar to Fig. 2B) for each ROI were normalized and averaged, and then fit to a single or double exponential decay from which the decay rates were determined (R_1_ and R_2_, Equation 1). The decay rates were expressed in units of images^−1^ as the time to collect a single image varied slightly depending on the image acquisition settings. Decay rates were calculated with continuous laser illumination or with 250 ps wide pulses at 80, 50 or 20 MHz. Decay rates were normalized using the continuous laser illumination so data sets from different experimental conditions could be compared. As expected, the rate of photo-bleaching decreased with pulsed illumination (Fig. 3A). The decay rate continued to decrease as the overall light exposure was reduced at slower pulse rates (Fig. 3A).

To determine if this reduced photo-bleaching was due to laser pulsing or simply a result of the reduced total light exposure of the sample, experiments were conducted with variable experiment times. This corresponded to the schematic of Case 2 in Fig. 1. To achieve this variable time the scan speed of the CLSM was changed to ensure that the sample was exposed to light for a total time of 1 μs at each pixel location. The pixel dwell time was 1 μs for continuous illumination, 12.6 μs, 25.2 μs and 50.4 μs for 80 MHz, 50 MHz and 20 MHz pulsing rates respectively. When pulsing the laser light equal light exposure resulted in 50% lower bleaching decay rates versus continuous illumination (Fig. 3B). However, the time between the 250 ps pulses did not have an effect on the decay rate (Fig. 3B, compare 80, 50 and 20 MHz pulsing rates). Thus, the pulsing of the laser light on the nanosecond time scale reduced photo-bleaching. However, the continued decrease in bleaching rates seen with slower pulsing rates is due to reduced overall light exposure not the time delay between pulses (Fig. 3A, compare 80, 50 and 20 MHz pulsing rates).

### Microsecond Pixel Dwell Times Eliminate Rapid Photo-bleaching Processes

**Figure 4:**
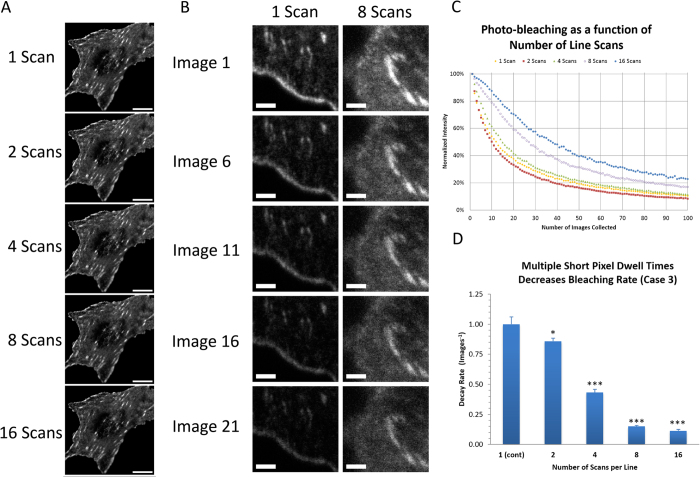
Rapid line scanning reduces photo-bleaching. (**A**) CHO-K1 cells expressing paxillin-EGFP were fixed with PFA and imaged with 2% power from the 488 nm laser on the CLSM with variable numbers of line scans. (**B**) Using 5% laser power and keeping the pixel dwell time constant but scanning rapidly 8 times or slowly 1 time. **(C**) Photo-bleaching curves for one slow scan or 2, 4, 8 or 16-line scans. (**D**) Average normalized decay rates in images^−1^ for various scan speeds and numbers. Scale bars are 10 μm in A and 2 μm in B. Error bars in (**D**) are standard error of the decay fitting. P values were calculated using a t-test of means and are shown relative to the 1-scan setting. *represents P < 0.05 and ***P < 0.001.

Researchers do not routinely have access to lasers with 250 ps pulse widths that pulse on the nanosecond timescale (i.e. 80, 50, 20 MHz). However, for imaging of fluorescent proteins CLSMs are typically equipped with continuous illumination from gas or solid state lasers. The line scanning feature of the CLSM can be used to simulate a “pulsing” of the laser on the timescale of the pixel dwell time (Fig. 1, Case 3). Experiments were conducted using the line averaging feature of the LSM710. Variable numbers of line scans were averaged while keeping the total pixel dwell time constant at 12.5 μs (Table 1). The intensity of the sample image was independent of the number of line scans that were collected (Fig. 4A). This is expected since the total pixel dwell time for each image is the same. However, when the sample was scanned successively over time, the use of rapid scanning and line averaging reduced photo-bleaching when compared to imaging with one slow scan. As such the EGFP intensity was much more stable over time when images were collected using 8 rapid line scans (pixel dwell time of 1.58 μs) versus one slow scan (Fig. 4B). A complete set of quantitative experiments was conducted with fixed CHO-K1 cells expressing paxillin-EGFP. Cells were mounted in PBS without any anti-fade present. Three cells were chosen and three different 100 × 100 pixel ROIs were photo-bleached with continuous imaging using 5% laser power from the 488 nm laser line. Three ROIs within each 100 × 100 image were chosen for intensity decay analysis. The experiment was performed in triplicate for a total of 27 ROIs for each of the five different microscope configurations. ROI intensity values were corrected for background and normalized. Photo-bleaching decay curves for each number of line scans were averaged and decay curves were fit to a one or two component exponential decay function (See Equation 1 in Methods). Photo-bleaching was progressively slower with more rapid scan speeds (Fig. 4C). The decay curve from a single slow scan, 2-line scans or 4-line scans were best fit to a double exponential decay with the fast decay rate on the order of 10–20 image frames and a slower decay time of 40–55 image frames (Fig. 4C). However, when rapidly scanning with 8-line or 16-line averages the fast photo-bleaching component was absent and the slower component was on the order of 80 image frames. Decay rates for the fast component (1, 2-line or 4-line) or the slow component (8-line and 16-line averages) were normalized to the single slow scan rate and showed a significant decrease in photo-bleaching as the pixel dwell time was decreased (Fig. 4D). Note that the offset of the decay curves was representative of the total amount of EGFP that was not photo-bleached, and as expected this increased as the pixel dwell time was decreased (Fig. 4D). Experiments were repeated in the absence or presence of the oxygen scavenger, OxyFluor and its substrate DL-Lactate. In this case, the rate of photo-bleaching was very slow and was independent of the scan speed or number (Supplemental Fig. 1).

In order to demonstrate that this was not a phenomenon limited to EGFP similar experiments were conducted with CHO-K1 cells expressing mCherry paxillin. As with EGFP, mCherry showed a significant reduction in photo-bleaching (~50%) with rapid line scanning (Supplemental Fig. 2).

#### Widefield versus Spinning Disk Photo-bleaching

In order to demonstrate that this effect is not unique to the CLSM, experiments were conducted for widefield versus spinning disk confocal illumination. Spinning disk illumination is “pulsed” as each location in the sample receives laser illumination light periodically as pinholes in the spinning disk pass a location. In order to equalize total light exposure to the sample the exposure time of 15 ms for the wide field illumination (Supplemental Fig. 3A) versus 1000 ms for the spinning disk (Supplemental Fig. 3B). With the spinning disk illumination the rate of photo-bleaching of paxillin-EGFP was reduced by a factor of two (Supplemental Fig. 3C). Note that these experiments were conducted on the Diskovery TIRF/SD platform (Spectral Applied Research, Richmond, ON) which does not include a microlens array to focus the laser light on the pinholes. Thus, results may differ for the Yokogawa (Tokyo, Japan) microlens based spinning disk confocal microscopes.

### Rapid Line Scanning Reduces Cell Stress

**Figure 5:**
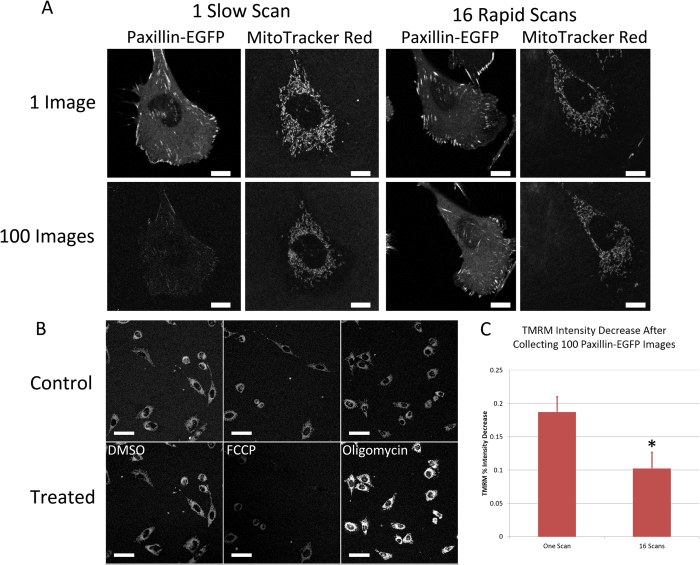
Rapid line scanning and mitochondrial morphology and membrane potential. (**A**) Live CHO-K1 cells expressing paxillin-EGFP were stained with MitoTracker Red^TM^. The mitochondrial morphology was imaged with the 543 nm laser at 1% power before and after 100 consecutive image scans of paxillin-EGFP were collected with the 488 nm laser line at 20% power. (**B**) TMRM controls with DMSO loading control, FCCP to depolarize the mitochondrial membrane or oligomycin to hyper-polarize the mitochondrial membrane. (**C**) Percentage decrease in 3D TMRM intensity following exposure of each cell for 100 images with the 488 nm laser line at 20% power. Scale bars are 10 μm in A and 50 μm in B. Error bars in (**C**) are SEM. One *represents P < 0.05.

It is logical to assume that reduced photo-bleaching will also result in reduced photo-toxicity in living samples. However, it is important to have assays that will assess light induced photo-toxicity. Visualization of mitochondrial morphology is a good metric because mitochondrial fission from a complex extended network into small vesicular mitochondrial fragments is an early indicator of cell stress^25^. Live CHO-K1 cells expressing paxillin-EGFP were used to determine if multiple rapid scans of high power laser light was less photo-toxic than using a single slow scan. MitoTracker Red was used as a Mitochondrial marker and the morphology was examined before and after exposure of the cells to 100 continuous scans with 20% laser power from the 488 nm laser line of a 25 mW argon ion laser. Although it is clear that 16 rapid line scans reduced photo-bleaching of paxillin-EGFP relative to one slow scan (Fig. 5A) there was no clear visual difference in mitochondrial morphology between the two conditions (Fig. 5A). There was some photo-bleaching of the MitoTracker Red probe but the mitochondria still showed an extended morphology, limited fission and remained dynamic regardless of the exposure of the cell to 100 image scans with the 488 nm laser and 16-line rapid scanning or 1-line slow scanning.

Tetramethyl rhodamine methyl ester (TMRM) is a very sensitive mitochondrial probe that changes intensity depending on mitochondrial membrane potential. Disruption of mitochondrial membrane potential is an early indicator of cell stress. The probe was validated by treating cells with 2 μM of carbonyl cyanide-4-(trifluoromethoxy) phenylhydrazone (FCCP) to depolarize the mitochondrial membranes or 2 μg/mL of oligomycin to hyperpolarize them. As expected, in the DMSO control the intensity of TMRM remained constant, following treatment with FCCP the intensity decreased and following treatment with oligomycin the intensity increased (Fig. 5B)^26^.

TMRM intensity was measured in CHO-K1 cells expressing paxillin-EGFP after the continuous imaging of 100 frames at 20% laser power with 1-scan slow imaging or 16-scan rapid imaging. The TMRM intensity was reduced with rapid line scanning. Note that the difference was only significant at 95% confidence (Fig. 5C). This was a complex measurement because the mitochondria morphology changes drastically over time and the structure is very dynamic in living cells. To minimize variability in the data TMRM was imaged in 3D using low laser power and the total intensity of the mitochondrial network was measured using Imaris software (Andor Technologies, Belfast, Ireland). Despite the significant decrease in mitochondrial intensity this assay is not ideal because changes in mitochondrial morphology and the need for high speed imaging in 3D make it difficult to obtain accurate intensity data over time.

### Cell Protrusion Increases and Cell Retraction Decreases with Rapid Line Scanning

**Figure 6:**
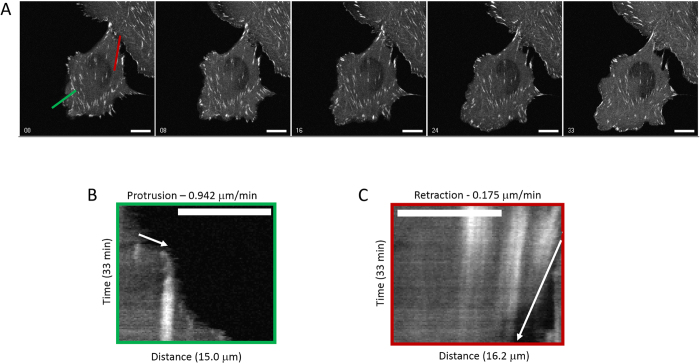
Cell protrusion and retraction rates measured using kymographs. CHO-K1 cells expressing paxillin-EGFP were imaged 100 times with continuous exposures to 1% laser power from the 488 nm laser line with single slow scans or 16 rapid scans. Cells were then imaged every 20 seconds for 33 minutes with low laser power. (**A**) Kymographs of intensity versus time were generated along protruding (green line) or retracting (red line) regions of the cell. (**B**) Intensity versus time Kymograph of cell protrusion along the green line shown in (**A**). (**C**) Intensity versus time Kymograph of cell retraction along the red line shown in A. Scale bars are 10 μm.

**Figure 7:**
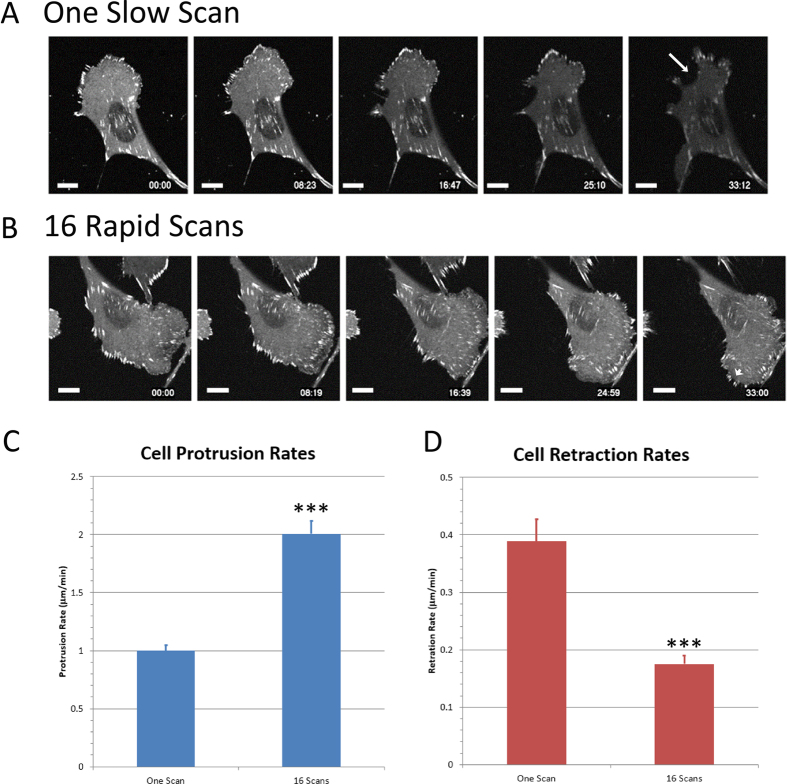
Cells protrude more and retract less with rapid line scanning. Live CHO-K1 cells expressing paxillin-EGFP were imaged 100 times consecutively with 1% laser power. Cells were then imaged every 20 s with 1% laser power for 33 minutes. (**A**) The 100 images were collected using 1-line slow scanning. Retraction of the cell is shown with the white arrow in image panel 5. (**B**) The 100 images were collected using 16-line rapid scanning. Protrusion is shown with the white arrowhead in image panel 5. Scale bars are 10 μm. Kymograph analysis was used to measure (**C**) cell protrusion rates and (**D**) cell retraction rates following exposure to 100 image scans with 1-line slow scanning or 16-line rapid scanning. Rates for multiple cells were calculated, averaged and plotted. Error bars are SEM. A t-test of means was conducted and three ***represents P < 0.001.

Based on our extensive experience with imaging of live CHO-K1 cells, imaging cell migration, and cell protrusion on 2D surfaces we have observed that the cells are highly sensitive to light. When CHO-K1 cells are exposed to high light levels they quickly begin to retract and “round up” off the substrate. The phenomenon makes the measurement of CHO-K1 cell protrusion and retraction rates an ideal assay for the study of subtle cell stresses. Live CHO-K1 cells expressing paxillin-EGFP were imaged every 20 s for 33 minutes with 1% laser power using either 1-scan slow imaging or 16-scan rapid imaging settings. Cell protrusion rates and retraction rates were measured using a kymograph analysis of cell edges that were undergoing protrusion (Fig. 6A, green line, 6B) and retraction (Fig. 6A, red line, 6C). Even under these “gentle” imaging conditions (~11 μW laser power, Table 2) it was clear that the one slow scan settings resulted in more photo-bleaching of paxillin-EGFP and also cell retraction (Fig. 7A, white arrow in image panel 5). When cells were imaged with the 16-scan rapid image settings there was little photo-bleaching and cells were highly dynamic and protrusive (Fig. 7B, white arrowhead in image panel 5). A quantitative kymograph analysis (Methods, Fig. 3) was used to measure cell protrusion and retraction rates during exposure to slow or rapid scan settings. It is clear that using rapid 16-line scanning results in more protrusive (Fig. 7C) and less retractive (Fig. 7D) cells. This is a very sensitive assay that shows that reduced photo-bleaching translates into reduced photo-toxicity with low laser powers and rapid line scanning with the CLSM.

Brightfield imaging was used in order to measure the “normal” protrusion and retraction rates of CHO-K1 cells. WT-CHO-K1 cells or CHO-K1 cells expressing paxillin-EGFP had protrusion rates of ~2 μm/min (Supplemental Fig. 4A) and retraction rates of ~0.75 μm/min (Supplemental Fig. 4B). If WT-CHO-K1 cells were exposed to bright field and blue excitation light that would normally be used to excite paxillin-EGFP fluorescence the cells still demonstrated “normal” protrusion and retraction rates (Supplemental Fig. 4). However, once the process of fluorescence excitation and emission of paxillin-EGFP was initiated protrusion rates were reduced and retraction rates were increased (Supplemental Fig. 4).

## Discussion

With the array of available tools, live cell fluorescence imaging is now commonplace and spans research fields in the physical, life and health sciences. However, most cells are never exposed to light during their lifetime and it is well known that high levels of light can induce toxic effects on living systems. Therefore, it is important to understand how light is delivered to the living specimen and how that may affect cell health. In this study, fixed and live cell assays were used to show that engineering the method of light delivery to the specimen in time can have a drastic effect on fluorophore photo-bleaching and live cell photo-toxicity.

It was not surprising that maintaining the same length of experimental time and pulsing a light source reduces photo-bleaching. This is primarily because the sample is only exposed to light during part of the experimental time (Fig. 1, Case 1, Fig. 3A) so the overall light exposure is reduced. This reduction scales with the amount of time the light is on (Fig. 3A). Pulsing the light on the nanosecond time scale resulted in an approximately 50% decrease in the photo-bleaching rate of EGFP. However, the rate of bleaching was not dependent on the time delay between the pulses (Fig. 3B). In general, most photo-bleaching is due to molecules in the triplet state absorbing additional light energy and generating oxygen radicals as they degrade. In this experimental system the time between light pulses was on the nanoseconds time scale (12.5 ns, 20 ns or 50 ns). This timescale is very short compared to the microsecond lifetime of the EGFP triplet state. Therefore regardless of whether pulses are 12.5, 20 or 50 ns apart the probability of a triplet state molecule absorbing a second photon on this time scale is very small. This is likely why the decay rate of EGFP bleaching is independent of the nanosecond laser pulsing rate (Fig. 3B).

Many researchers do not have access to nanosecond pulsed lasers but do have access to a CLSM. The line scanning feature of the CLSM can be used as a method to “pulse” the laser light on the sample. Experiments on the CLSM could be conducted with pixel dwell times that were as short at 0.79 μs. The data presented here showed that multiple rapid scans of the laser eliminated a fast bleaching component of EGFP and reduced the photo-bleaching rate by almost nine fold (Fig. 4D) without any impact on image quality (Fig. 4A). A more than two-fold reduction in the photo-bleaching rate of mCherry was also observed (Supplemental Fig. 2). Thus, the application of rapid line scanning will likely yield similar reductions in photo-bleaching for many other fluorophores and fluorescent proteins. This is a tremendous opportunity for every researcher using a CLSM to seize. For fixed cell imaging photo-bleaching will be minimized and will be especially helpful for high resolution imaging of large 3D samples with multiple fluorphores. For live samples this will most importantly result in a reduction in photo-toxicity. The technique can be implemented very simply by changing the software settings to multiple fast line scanning (i.e. shorter pixel dwell times) that will preserve samples and with line averaging will not impact the image quality. With the advent of tissue clearing and rapid super-resolution techniques reduced photo-bleaching will be important for high resolution imaging of large samples and for 3D time-lapse imaging of living samples.

The pixel dwell time for 8-line and 16-line scan settings is shorter than the lifetime of the triplet state of EGFP. Therefore, it is likely that the drastic reduction in photo-bleaching is related to a low probability of triple state molecules to absorb additional light energy with microsecond or sub-microsecond light pulsing. The time between line scans was on the millisecond timescale giving any triplet state molecules adequate time to relax back to the ground state before the next rapid line scan. The involvement of the triplet state in the photo-bleaching process is supported by the minimal photo-bleaching measured in the presence of oxygen scavengers (Supplemental Fig. 1). More work needs to be done to determine if each fluorophore will have an ideal pixel dwell time for a given laser power to minimize photo-bleaching.

Many other methods of light delivery to the sample, aside from the standard CLSM, could possibly reduce photo-bleaching as well. Similar experiments comparing widefield and spinning disk illumination showed that reduced photo-bleaching can result from the spinning disk “pulsing” with a different microscope architecture of light delivery to the sample (Supplemental Fig. 3). Additionally, theory shows that the resonant scanning CLSM should see similar reductions in photo-bleaching and photo-toxicity^21^. To our knowledge there has not been a systematic laboratory study of these systems. Given the results shown here further studies will most likely demonstrate that the resonant scanning CLSM will show similar reductions in fluorophore photo-bleaching and cellular photo-toxicity.

The live cell assays presented here, specifically the cell protrusion assay, demonstrates that moving to rapid line scanning also results in reduced photo-toxicity. Thus, for any researcher performing live cell CLSM experiments they should immediately move away from single slow line scanning to averaging of multiple rapid line scans. Even under relatively “gentle” imaging conditions with 1% laser power (~11 μW) a significant reduction in photo-bleaching and improved cell movement can be seen when moving from slow line scanning to rapid line averaging (Fig. 7).

These studies show how a careful and systematic study of how light is delivered to the sample can result in marked reductions in photo-damage without any compromise in image quality. However, these studies have just begun to explore the possibilities of engineering light delivery to the sample in space and time such that they minimize hazardous sample conditions and maximize experimental output. Modern microscopes come with a plethora of designs and choosing how the light is delivered to the specimen could be critical for reproducible and accurate live cell experiments. The protrusion assay is straightforward to implement and should prove useful as a metric for photo-toxicity of any fluorescence microscopy technique. It would be interesting to apply the technique to a more in depth study of spinning disk confocal microscopy, resonant confocal laser scanning microscopy, light sheet microscopy^27^ and the super-resolution techniques. As live cell applications become more and more prevalent, the tools presented here will be essential to ensure procedures and protocols are optimized so the processes under investigation are not unduly influenced by fluorescence imaging.

## Methods

### Sample Preparation

Fibronectin (Sigma, F-0895) coating of 35 mm coverglass bottom dishes (World Precision Instruments, FD-35) was conducted by diluting a 10 μL aliquot of 1 mg/mL stock solution with PBS and placing 1 mL of 2 μg/ml solution on 35 mm dishes for either one hour at 37 °C or overnight at 4 °C. Dishes were then gently washed three times with PBS. Coated dishes were used immediately or stored at 4 °C in PBS solution for up to two weeks. Chinese hamster ovary K1 (CHO-K1) cells stably expressing paxillin-EGFP or paxillin-mCherry were used for all of the studies. Cells were grown in low glucose DMEM (ThermoFisher Scientific, Grand Island, NY, 11885-084) supplemented with non-essential amino acids (ThermoFisher Scientific, 11140-050), 1% penicillin-streptomyosin (ThermoFisher Scientific, 10378-016), 10% fetal bovine serum (ThermoFisher Scientific, 10082-147) and 25 mM HEPES (ThermoFisher Scientific, 15630-080). The media also contained 0.5 mg/mL of geneticin for maintenance of selection of paxillin-EGFP or paxillin-mCherry expressing cells (ThermoFisher Scientific, 11811-031). For fixed cell experiments, cells were washed twice with PBS and fixed in a solution of 4% paraformaldehyde (PFA) for 15 minutes at room temperature. A solution of 16% PFA (Polysciences, Warrington, PA, 50-00-0) was diluted to 4% with PBS and used immediately or frozen at −20 °C for later use. PFA solution should not be stored at room temperature as it breaks down rapidly leaving highly fluorescent byproducts.

For fixed cell photo-bleaching experiments, cells were placed in PBS solution with no anti-fade or anti-bleaching agents. Fixed cells were stored at 4 °C and used over a period of 4–6 weeks with no visible signs of deterioration.

For live cell experiments cells were maintained in exponential growth conditions and lifted with trypsin and plated on 35 mm fibronectin coated cover glass bottom petri dishes and left to grow overnight before experimentation. For cell protrusion experiments and mitochondrial morphology imaging, cells were labeled with 100 μM MitoTracker Red CMXRos (ThermoFisher Scientific, M-7512) for 30 minutes at 37 °C immediately before experimentation. MitoTracker Red solution was removed, cells were washed three times with full-DMEM and then 2 mL of full-DMEM was placed on the cells before imaging.

### Image Collection on the CLSM

Images were collected using a Zeiss 710 CLSM with a fully automated inverted Axio Observer microscope using a plan-apochromat 63x/1.4 NA oil immersion objective lens. Cells were placed in a stage top microscope incubation chamber (Chamlide, Live Cell Instrument, Seoul, Korea) at 37 °C with 5% humidified CO_2_. Cells were located and focused using brightfield imaging. Samples were then observed visually with the microscope eyepieces using an EXFO lamp as the illumination source. To avoid photo-bleaching or photo-toxicity during visualization the lamp was set at 12% power and a 1% neutral density (ND) filter was put in the light path. This resulted in an incident power of ~0.12% at the sample. The system was set up for imaging using a single band dichroic beam splitter and the 473 nm pulsed laser or the 488 nm laser line from a 25 mW argon ion laser. The emission bandpass was set to 493 nm–598 nm. Images of 512 × 512 pixels were collected at zoom 2.0 or zoom 3.0 with a pixel size of 0.17–0.088 μm. Images were collected and saved as 12-bit. lsm files. The pinhole size was set to 1–2 Airy units, the detector gain to 700–800, the digital offset to 10–15 (to avoid pixels reading 0 intensity units) and the digital gain of 1.0 in the uni-directional line-scanning mode. Prolong^®^ Gold anti-fade mounting media (ThermoFisher Scientific, P36934) was only used for fixed cell intensity comparisons to avoid photo-bleaching during repeated imaging of the same cell. For photo-bleaching experiments, a 100 × 100 pixel region of interest (ROI) (8.70 μm × 8.70 μm) was selected and a time series of 100–300 images of that region was collected with continuous scanning (i.e. no time delay between frames).

### Photo-bleaching Experiments

Three scenarios were created for studying the effects of continuous or pulsing of the laser using the 63x/1.4 NA objective lens (Fig. 1). For nanosecond pulsing, images were collected with a 473 nm laser in continuous mode or pulsed with 0.25 ns (250 ps) pulses at 80 MHz (12.5 ns between pulses), 50 MHz (20 ns between pulses) or 20 MHz (50 ns between pulses). Unless otherwise noted, the scan speed was set to 2 (6.30 μs/pixel) with one single scan and the detector gain was set to 700. Other settings were the same as above. **Case 1:** The total duration of the experiment remained constant and the 473 nm laser power was set to 40%. Images were collected with continuous mode or pulsing at 20, 50 or 80 MHz. This resulted in a lower light exposure for samples as the pulsing rate decreased (i.e. fewer pulses of light over the fixed experimental time) **Case 2:** The 473 nm laser power was kept constant at 100% but the total light dosage at each pixel location was kept the same. This was achieved by increasing the pixel dwell time so that the sample received a constant total light dosage with each pass of the laser. The pixel dwell time was 1 μs for continuous illumination, 12.6 μs for 80 MHz pulsing, 25.2 μs for 50 MHz pulsing and 50.4 μs for 20 MHz pulsing. **Case 3:** These experiments used the standard CLSM 488 nm laser line (EGFP) from an argon ion gas laser in continuous mode (i.e. no pulsing) or the 543 nm laser line (mCherry). “Pulsing” of the laser was simulated using the multiple line scanning feature of the microscope. Each pixel location in the sample was either exposed to light once for a long period of time (i.e. one scan) or multiple times for a shorter period of time (2, 4, 8 or 16 scans). The total time each pixel location within the sample was exposed to light was held constant. In this case, the time between “pulses” was on the millisecond scale corresponding to the line scan time of the system. The single scan is similar to continuous illumination at each pixel with the 473 nm laser. The pulse duration (i.e. pixel dwell time) was controlled with variable scan speeds and multiple passes of the sample were used to keep the total light dosage at each pixel (i.e. pixel dwell time) constant (Table 1). This ensured the total light dosage at each pixel was of the same peak power and the same total time. The total image collection time was set to a constant of 1.51 seconds and 100 images were collected in the experimental time of 2 minutes and 31 seconds. The variable scanning conditions are shown in Table 1. Oxygen depletion EGFP-paxillin fixed cell experiments were carried out using a 1:100 dilution of OxyFlour (Oxyrase Inc., OF-0005) in PBS with 20 mM DL-Lactate (Sigma Aldrich, 71720).

For all experiments, small ROIs of paxillin-EGFP expression in the cell were imaged for 100 or more frames and intensity over time data was measured for several ROIs within the dataset (Fig. 2A,B). Data sets for multiple ROIs were saved in Excel, corrected for background intensity and pooled for each experimental condition.

### Spinning Disk versus Widefield Photo-bleaching

Spinning disk photo-bleaching of fixed paxillin-EGFP cells was compared to wide field excitation. Experiments were conducted using a Diskovery TIRF/SD microscope (Quorum Technologies Inc.). Widefield excitation was performed with 10% power of the 488 nm laser with the spinning disk removed from the light path. An exposure time of 15 ms was used for imaging with stream acquisition in MetaMorph Version 7.8.12.0 using the 63x/1.47 NA Leica (Wetzler, Germany) objective lens and a QuantEM-CCD camera (Hamamatsu, Japan). To keep the light dose constant the exposure time was adjusted with the 50 μm pinhole disk in place to keep the average image intensity constant with that of the widefield image. This resulted in a 1000 ms exposure versus the 15 ms exposure with wide field “continuous” imaging.

### Photo-bleaching Image Analysis

Photo-bleaching data analysis was conducted using the Zen 2009 or MetaMorph software. ROI intensities were extracted as text files and plotted versus time for each image acquisition configuration. Background intensity values were subtracted from each ROI photo-bleaching data set. To properly compare data sets collected with different experimental times the intensity values were plotted versus the number of images collected rather than the time to collect the images. Intensity data was then normalized to the highest intensity timepoint and the photo-bleaching data for all ROIs from multiple experiments were averaged. The background corrected and normalized intensity results were then plotted (Fig. 2B) and fit to a single or double exponential decay (Equation 1) using SigmaPlot software (Systat Software, Inc., San Jose, CA) or Origin software (Northampton, MA). Where *a*_*1*_ and *a*_*2*_ are the relative amplitudes of each decay component and *R*_*1*_and *R*_*2*_ are the decay rates for each component.


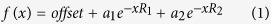


For easier comparison between experiments performed with different laser powers or different laser lines (e.g. 473 nm vs 488 nm), photo-bleaching rates were expressed in terms of the number of images collected and were normalized to the continuous illumination dataset within each experiment. For comparison between different lasers and different experimental settings the laser powers used for the different experiments are shown in Table 2.

For the line scan experiments all of the decay curves were fit with high R squared values (R^2^ > 0.99). Fit values for rate constants were very reproducible with low standard deviations of 1–7% between experiments and ROIs. The offset (y_o_), or amount of fluorescence intensity that was not photo-bleached at the end of the experiment, was found to increase as the pixel dwell time decreased with 5–10% unbleached with pixel dwell time of 3–13 μs and 15% unbleached with pixel dwell times of 0.8–1.6 μs

### Mitochrondrial Morphology and TetraMethyl Rhodamine Methyl Ester (TMRM)

CHO-K1 cells expressing paxillin-EGFP were stained with MitoTracker Red CMXRos (ThermoFisher Scientific, M-7512) using the manufacturers protocol. Cells were exposed to 488 nm laser light for 100 continuous scans at 20% laser power. Exposure was conducted with one single slow line scan or 16 rapid line scans that were averaged. Mitochondrial morphology was imaged in 3D. The z-stack of images of the MitoTracker stain was collected with 1% laser power from a 2 mW-543 nm laser line. Approximately 20 images at 0.36 μm apart were collected for each cell.

Mitochondrial membrane potential was imaged using TMRM staining. TMRM is a cell-permeant, cationic, red-orange fluorescent dye that is readily sequestered by active mitochondria. TMRM accumulates in the inner membrane of mitochondria in healthy cells, and is released into the cell cytosol when the membrane potential depolarizes during apoptosis^28^. Therefore, high TMRM is an indication of cell health and cell stress results in a decrease in TMRM intensity. TMRM experimental conditions were validated with 2 μM carbonyl cyanide-4-(trifluoromethoxy)phenylhydrazone (FCCP; Sigma Aldrich, Milwaukee, WI) to depolarize cells and decrease TMRM staining or 2 μg/mL oligomycin (Sigma Aldrich, Milwaukee, WI) to hyperpolarize the mitochondria and increase TMRM staining. TMRM solid powder (ThermoFisher Scientific, T-668) was dissolved in DMSO to make a stock solution of 5 mg/mL. The TMRM stock solution was then diluted to 20 μM with complete DMEM cell culture media and applied to paxillin-EGFP expressing CHO-K1 cells in a 35 mm glass bottom dish (prepared as described above) at 37 °C and allowed to adhere overnight. Cells were then washed two times with DMEM and left in 2 mL of fresh DMEM for live cell imaging. Cells were exposed to 488 nm laser light for 100 continuous scans at either 1% or 20% power. Exposure was conducted with one single slow line scan or 16 rapid line scans that were averaged. Both before and following exposure to 488 nm light 3D image-stacks of the TMRM staining were collected. Images of the TMRM were generated with 3% power from a 2 mW-543 nm laser line. The main dichroic beam splitter was set to MBS 488/543 to reflect both laser lines. Transmitted light images were also collected. The transmitted light detector (T-PMT) was set to a gain of 180 and a digital offset of 15. TMRM intensities were measured using the surface analysis feature in the Imaris software package. The “surface detail” was set to 0.1 μm and thresholding with the “diameter of the largest sphere which fit into the object” set to 0.5 μm. The intensity of the entire mitochondrial volume stained with TMRM was then measured and output to Excel. The change in intensity from before to after imaging with 488 nm laser light was then calculated for each cell and averaged for each condition.

### Cell Protrusion and Retraction

Live cell experiments were conducted on CHO-K1 cells expressing paxillin-EGFP. Cells were exposed to 100 sequential image scans with 488 nm laser excitation at 1% with imaging settings of one slow line scan and a 12.61 μs pixel dwell time, or with 16-scan line averaging with a 0.79 μs pixel dwell time for a total pixel dwell time of 12.64 μs (Table 1). Cells were then imaged every 20 s for 33 minutes to observe cell protrusion and retraction (Fig. 6A). Multiple cells within the sample were imaged for each condition. Cell protrusion and retraction rates were measured using a kymograph analysis performed using MetaMorph software (Molecular Devices, Sunnyvale, CA) (Fig. 6B). To analyze these rates, a line was placed at a 90° angle from the leading or trailing edge of the cell from which the intensity along that line was plotted versus time for protruding (green line, Fig. 6A,B) or retracting (red line, Fig. 6A,C) regions of the cell. Rates were measured from the slope of the cell edge in the kymograph plots (white arrows, Fig. 6B,C).

Cell protrusion measurements were also conducted using brightfield imaging with an inverted Zeiss Axiovert 200M microscope equipped with a HAL 100 halogen light source, an Axiocam 506 monochrome camera and a 20x/0.8 NA objective lens. Exposure times of 150–300 ms were used to acquire all images.

## Additional Information

**How to cite this article**: Boudreau, C. *et al*. Excitation Light Dose Engineering to Reduce Photo-bleaching and Photo-toxicity. *Sci. Rep*. **6**, 30892; doi: 10.1038/srep30892 (2016).

## Supplementary Material

Supplementary Information

## Figures and Tables

**Table 1:** Summary of Line Scanning Settings.

Configuration	Software Scan Speed Setting	Number of Lines Scanned	Pixel Dwell Time	Total Pixel Dwell Time (Pixel Dwell Time x Number of Lines Averaged)
1	5	1	12.61 μs	12.61* 1 = 12.61 μs
2	6	2	6.30 μs	6.30* 2 = 12.60 μs
3	7	4	3.15 μs	3.15* 4 = 12.60 μs
4	9	8	1.58 μs	1.58* 8 = 12.64 μs
5	12	16	0.79 μs	0.79* 16 = 12.64 μs

Line scan settings using the standard CLSM and the 488 nm laser at 5% power. The total image acquisition time was 1.51 s while the number of line scans and the pixel dwell time were varied to mimic laser pulsing. The “pulse width” was essentially the pixel dwell time and the number of pulses was the number of line scans. The time between pulses was on the milliseconds time scale and was related to the time for the laser to scan back to the start of the line between line scans.

**Table 2:** Laser percentage to power conversions.

Laser Line (nm)	Power (%)	Average Power (μW)	Experiments that used this power	Figures where this power was used
473	40	500	ns pulsing	Figure 3
473	5.6	67	ns pulsing	Figure 3
488	1	11	Protrusion-retraction Assay	Figure 6
	2	22	Imaging	Figures 4 and 7
	5	50	Variable line scanning, EGFP-Blue Excitation	Figure 4, Supplemental Figure 4
	5.6	57	OxyFlour^TM^	Supplemental Figure 1
	20	260	TMRM and morphology of the mitochondria assays	Figures 5 and 7
543	1	4	TMRM Imaging	Figure 5
	25	97	mCherry Photobleaching	Supplemental Figure 2
488	10	184	Wide-field/Spinning disk	Supplemental Figure 3

Laser powers were measured using a Coherent FieldMaxII-TO laser power meter. Note that powers were measured with the 10x/0.3 NA lens and were not necessarily measured every time an experiment was conducted.

## References

[b1] Lippincott-Schwartz J. The long road: peering into live cells. Nat Cell Biol 12, 918 (2010). 20885416 10.1038/ncb1010-918

[b2] Lacoste J., Vining C., Zuo D., Spurmanis A. & Brown C. M. In Annual Reviews in Fluorescence 2010 (ed. Geddes C. D.) (2011).

[b3] Galdeen S. A. & North A. J. Live cell fluorescence microscopy techniques. Methods in molecular biology 769, 205–222 (2011). 21748678 10.1007/978-1-61779-207-6_14

[b4] Goldman R. D. & Spector D. L. Live Cell Imaging: A Laboratory Manual. (Cold Spring Harbor Laboratory Press, 2005).

[b5] Graf R., Rietdorf J. & Zimmermann T. Live cell spinning disk microscopy. Adv Biochem Eng Biotechnol 95, 57–75 (2005). 16080265 10.1007/b102210

[b6] Haraguchi T. Live cell imaging: approaches for studying protein dynamics in living cells. Cell Struct Funct 27, 333–334 (2002). 12502886 10.1247/csf.27.333

[b7] Swedlow J. R. & Platani M. Live cell imaging using wide-field microscopy and deconvolution. Cell Struct Funct 27, 335–341 (2002). 12502887 10.1247/csf.27.335

[b8] Wiedenmann J., D’Angelo C. & Ulrich Nienhaus G. In Fluorescent Proteins II: Applicaiton of Fluorescent Protein Technology Vol. 12 Springer Series on Fluorescence: Methods and Applications (ed Wolfbeis O. S.) 3–33 (Springer, 2012).

[b9] Frigault M. M., Lacoste J., Swift J. L. & Brown C. M. Live-cell microscopy-tips and tools. J Cell Sci 122, 753–767 (2009). 19261845 10.1242/jcs.033837

[b10] Waters J. C. Live-cell fluorescence imaging. Methods in cell biology 114, 125–150 (2013). 23931505 10.1016/B978-0-12-407761-4.00006-3

[b11] Editorial. Artifacts of Light. *Nature Methods* **10** (2013).

[b12] Carlton P. M. . Fast live simultaneous multiwavelength four-dimensional optical microscopy. Proceedings of the National Academy of Sciences of the United States of America 107, 16016–16022 (2010). 20705899 10.1073/pnas.1004037107 PMC2941331

[b13] Waldchen S., Lehmann J., Klein T., van de Linde S. & Sauer M. Light-induced cell damage in live-cell super-resolution microscopy. Scientific Reports 5, 15348 (2015). 26481189 10.1038/srep15348 PMC4611486

[b14] Magidson V. & Khodjakov A. Circumventing photodamage in live-cell microscopy. Methods in cell biology 114, 545–560 (2013). 23931522 10.1016/B978-0-12-407761-4.00023-3 PMC3843244

[b15] Hoebe R. A. . Controlled light-exposure microscopy reduces photobleaching and phototoxicity in fluorescence live-cell imaging. Nat Biotechnol 25, 249–253 (2007). 17237770 10.1038/nbt1278

[b16] Li Y. F. & Sancar A. Active site of Escherichia coli DNA photolyase: mutations at Trp277 alter the selectivity of the enzyme without affecting the quantum yield of photorepair. Biochemistry 29, 5698–5706 (1990). 2200511 10.1021/bi00476a009

[b17] Nishigaki T., Wood C. D., Shiba K., Baba S. A. & Darszon A. Stroboscopic illumination using light-emitting diodes reduces phototoxicity in fluorescence cell imaging. Biotechniques 41, 191–197 (2006). 16925021 10.2144/000112220

[b18] Donnert G., Eggeling C. & Hell S. W. Major signal increase in fluorescence microscopy through dark-state relaxation. Nat Methods 4, 81–86 (2007). 17179937 10.1038/nmeth986

[b19] Penjweini R., Loew H. G., Hamblin M. R. & Kratky K. W. Long-term monitoring of live cell proliferation in presence of PVP-Hypericin: a new strategy using ms pulses of LED and the fluorescent dye CFSE. J Microsc 245, 100–108 (2012). 21974829 10.1111/j.1365-2818.2011.03555.x PMC3232286

[b20] Ji N., Magee J. C. & Betzig E. High-speed, low-photodamage nonlinear imaging using passive pulse splitters. Nat Methods 5, 197–202 (2008). 18204458 10.1038/nmeth.1175

[b21] Borlinghaus R. T. MRT letter: high speed scanning has the potential to increase fluorescence yield and to reduce photobleaching. Microsc Res Tech 69, 689–692 (2006). 16878313 10.1002/jemt.20363

[b22] Visser A. & Hink M. A. New perspectives fluorescence correlation spectroscopy. Journal of Fluorescence 9, 81–84 (1999).

[b23] Widengren J., Mets U. & Rigler R. Photodynamic properties of green fluorescent proteins investigated by fluorescence correlation spectroscopy. Chemical Physics 250, 171–186 (1999).

[b24] Jimenez-Banzo A., Nonell S., Hofkens J. & Flors C. Singlet oxygen photosensitization by EGFP and its chromophore HBDI. Biophys J 94, 168–172 (2008). 17766345 10.1529/biophysj.107.107128 PMC2134865

[b25] Youle R. J. & van der Bliek A. M. Mitochondrial fission, fusion, and stress. Science 337, 1062–1065 (2012). 22936770 10.1126/science.1219855 PMC4762028

[b26] Gautier C. A. . Regulation of mitochondrial permeability transition pore by PINK1. Molecular neurodegeneration 7, 22 (2012). 22630785 10.1186/1750-1326-7-22 PMC3405481

[b27] Chen B. C. . Lattice light-sheet microscopy: imaging molecules to embryos at high spatiotemporal resolution. Science 346, 1257998 (2014). 25342811 10.1126/science.1257998 PMC4336192

[b28] Balzer E. M. . Physical confinement alters tumor cell adhesion and migration phenotypes. FASEB journal: official publication of the Federation of American Societies for Experimental Biology 26, 4045–4056 (2012). 22707566 10.1096/fj.12-211441 PMC3448771

