# Supplementary material for: Excitation Light Dose Engineering to Reduce Photo-bleaching and Photo-toxicity
Source: Sci Rep. 2016 Aug 3;6:30892. doi: 10.1038/srep30892 (PMC4971503; doi:10.1038/srep30892)
Supplement: Supplementary Information [file srep30892-s1.pdf]

EXCITATION LIGHT DOSE ENGINEERING TO REDUCE PHOTO-BLEACHING AND PHOTO-TOXICITY

Colton Boudreau[1,*], Tse-Luen (Erika) Wee[1,2,*], Yan-Rung (Silvia) Duh[3], Melissa P. Couto[3], Kimya H. Ardakani[4], and Claire M. Brown[1,2,¥]

1) Department of Physiology, McGill University
2) Advanced BioImaging Facility (ABIF), McGill University, 3649 Prom. Sir William Osler, Bellini Building Rm137, Montreal, QC, Canada, H3G 0B1
3) Department of Anatomy and Cell Biology, McGill University
4) Department of Microbiology and Immunology, McGill University

* Equal contribution by both authors.

¥ Corresponding Author: claire.brown@mcgill.ca

Supplemental Figure 1: Photo-bleaching of EGFP in the presence or absence of oxygen: Normalized CLSM photo-bleaching decay rates for paxillin-EGFP with one slow scan or 16-scan rapid scans in the presence of oxygen or with the oxygen scavenger OxyFluor™. T-tests were conducted and three *** corresponds to P<0.001. There was no significant difference between the decay rates for 1 scan and 16 scans in the presence of OxyFluor™. Error bars are SEM.

Supplemental Figure 2: Rapid line scanning reduces photo-bleaching: Fixed CHO-K1 cells expressing paxillin-mCherry imaged with 25% power from the 543 nm laser on the CLSM with variable numbers of line scans. **A)** Photo-bleaching curves follow a double exponential decay when scanning each line once slowly but a single exponential decay with averaging 16 rapid scans. **B)** Normalized average photo-bleaching decay rates for experiments that were conducted in triplicate with 9 cells and 15 ROIs for each condition. P values were calculated using a t-test of means and *** corresponds to P<0.001. Error bars are SEM.

Supplemental Figure 3: Reduced photobleaching with spinning disk confocal: Paxillin-EGFP in fixed CHO-K1 cells imaged **A)** with a 15 ms exposure time with wide-field laser illumination or **B)** with a 1000 ms exposure time with spinning disk confocal microscopy. **C)** Normalized photo-bleaching decay rates for fixed paxillin-EGFP with wide-field or spinning disk confocal excitation. The scale bars are 10 μm. Error bars are SEM.

Supplemental Figure 4: Photo-toxicity depends on EGFP fluorescence excitation and emission: Plots showing **A)** Cell protrusion rates and **B)** cell retraction rates. CHO-K1 cells lacking any EGFP protein fusion were imaged in bright field transmitted light (BF), or with blue fluorescence excitation light (Blue Excitation), cells expressing paxillin-EGFP but imaged only in BF (EGFP-BF) or with fluorescence excitation (EGFP-Blue Excitation). Images were collected every 20 seconds for 30 minutes in order to measure protrusion and retraction rates. T-tests of means relative to BF imaging and P<0.01 is shown at ** while P<0001 is ***. Error bars are SEM.

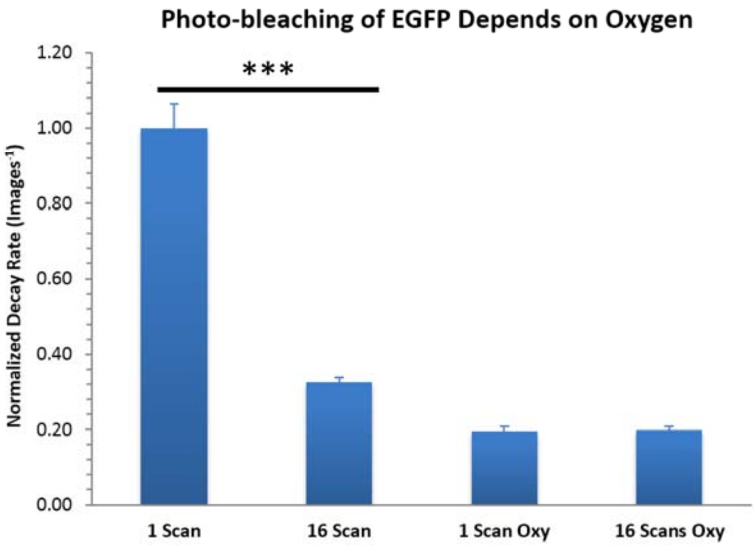

Supplemental Figure 1

A

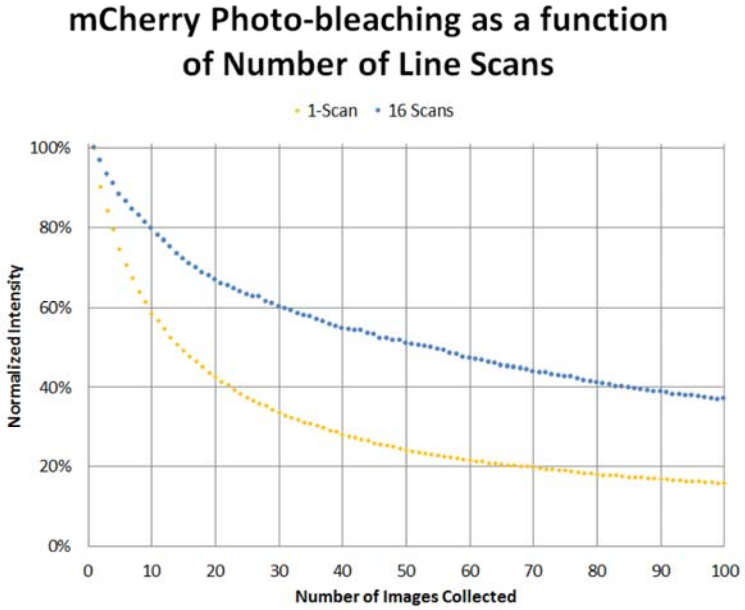

B

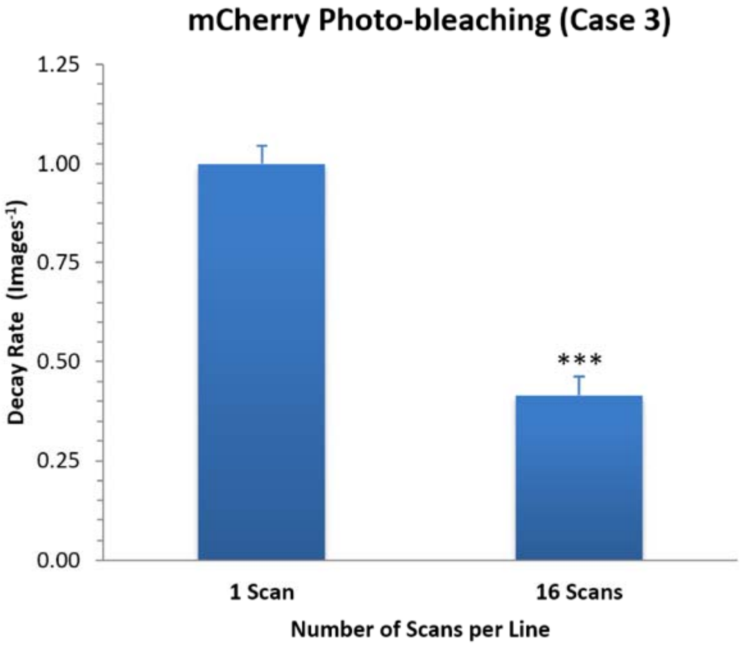

Supplemental Figure 2

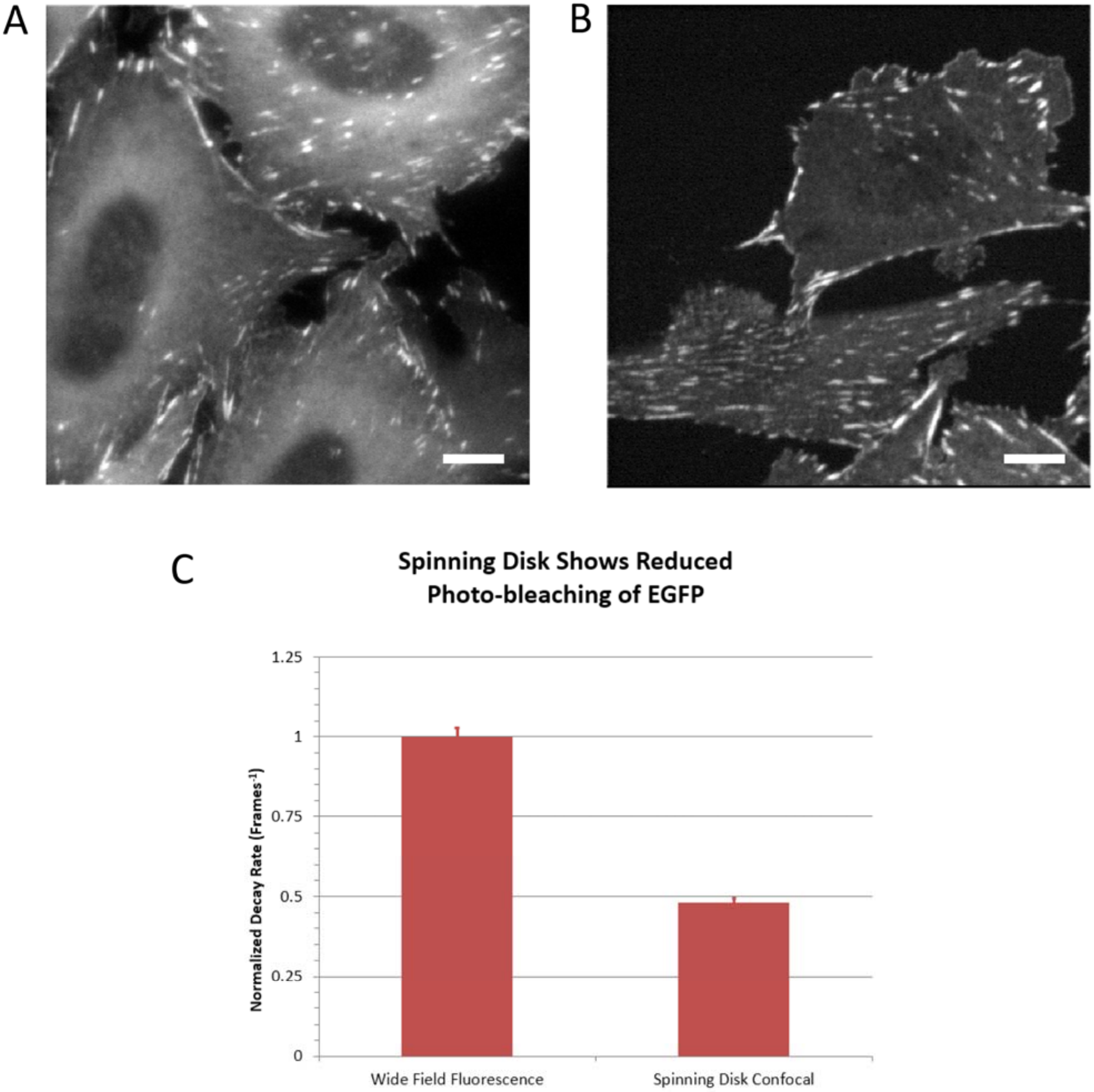

Supplemental Figure 3

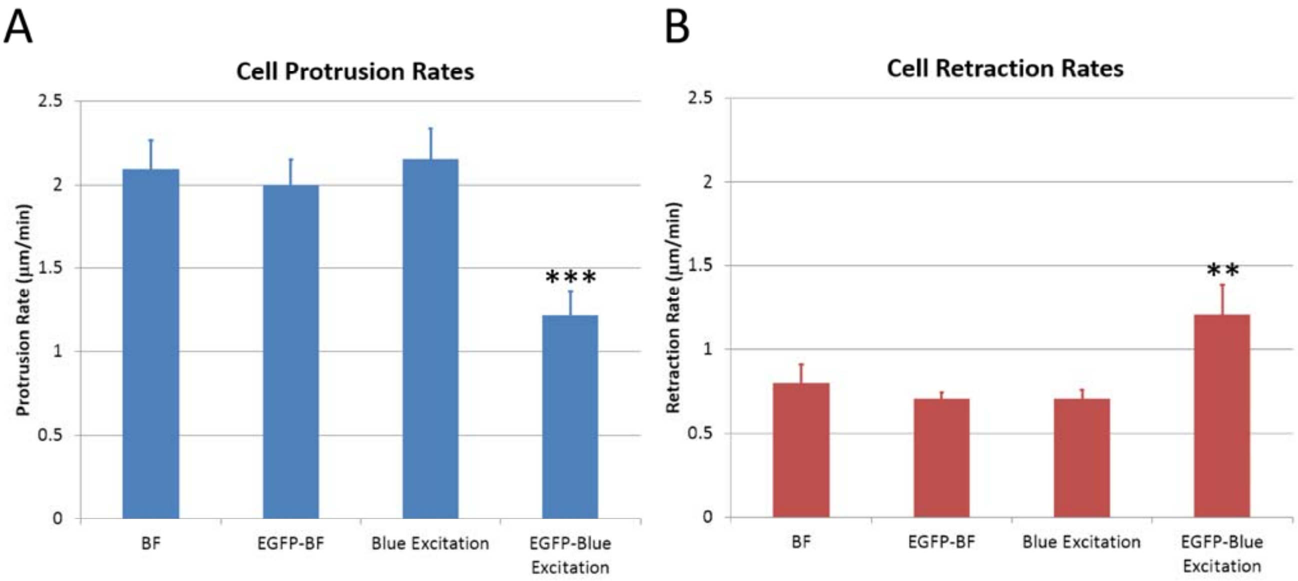

Supplemental Figure 4
